# Protective effects of asiaticoside on renal ischemia reperfusion injury in vivo and in vitro

**DOI:** 10.1080/21655979.2022.2061302

**Published:** 2022-04-17

**Authors:** Shengjie Tang, Xiangcheng Xie, Ming Wang, Lili Yang, Wei Wei

**Affiliations:** Department of Nephrology, Affiliated Hangzhou First People’s Hospital, Zhejiang University, School of Medicine, Hangzhou, Zhejiang, People’s Republic of China,310000

**Keywords:** Asiaticoside, renal ischemia-reperfusion injury, macrophage polarization, inflammatory mediators

## Abstract

Ischemia/reperfusion injury (I/R) is the main causes of acute kidney injury (AKI), which is a global health concern. Evidence suggests that asiaticoside plays vital roles on anti-inflammatory and, anti-kidney fibrosis effects, and promotes tissue repair. However, the effects of asiaticoside on AKI caused by ischemia-reperfusion have not been well defined. Herein, we explored the protective effect of asiaticoside on renal ischemia-reperfusion injury (IRI) using in vivo and in vitro studies, and elucidated the potential mechanism of asiaticoside-mediated repair. Results showed that asiaticoside attenuated the levels of blood urea nitrogen (BUN) and serum creatinine (Scr) in the IRI model. Meanwhile, asiaticoside reduced the secretion of IL-6, IL-1β and TNF-α, but increased IL-10 secretion in a dose-dependent manner. Treating Raw264.7 cells with lipopolysaccharide (LPS) induced an inflammatory response, but the LPS-induced effects were attenuated after administering asiaticoside. Furthermore, asiaticoside significantly inhibited the expression of inducible Nitric Oxide Synthase (iNOS) and promoted the expression of Arginase1 induced by LPS, which are the polarization marker proteins. In conclusion, this study shows that asiaticoside possesses protective action in AKI after ischemia-reperfusion, due to the inhibition of inflammatory mediators and promoting transformation of macrophages from M1 type to M2 type.

## Graphical Abstract

**Figure:**
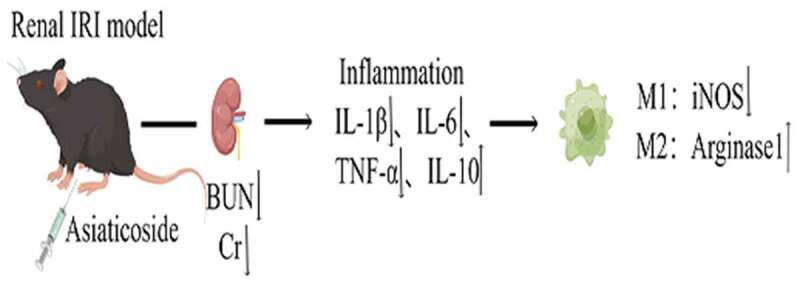

## 1. Introduction

Acute kidney injury (AKI), a common clinical syndrome defined by an abrupt decline in renal excretory function, has an incidence rate of about 5–15% in hospitalized patients [1]. Evidence suggests that even mild AKI is associated with a reduction in long-term survival [2]. This is because the onset of AKI can lead to the development of chronic kidney disease (CKD) or end-stage renal disease (ESRD) [3]. Ischemia-reperfusion injury (IRI) is one of the major causes of AKI, which induces microvasculature alterations, robust inflammatory responses, apoptosis, and necrosis [4]. However, the mechanism that regulates the repair process after AKI has not yet been fully elucidated.

Previous studies have suggested that inflammatory cascades of chemokines AND cytokines, the infiltration of macrophages and stress oxidative processes modulate the injury and repair stage of AKI [5–7]. It has also been reported that macrophages, as innate immune cells, have pathogenic roles in AKI [8,9]. Notably, mature macrophages respond to environmental signals through two polarization programs: classically activated macrophages (M1) and alternatively activated ones (M2) [10]. These two programs can be activated or inhibited by various cytokines, including Interleukin-6 (IL-6), Interleukin-1β (IL-1β) and Interleukin-33 (IL-33) [11,12]. Macrophages play an essential role in driving the progression or repair of IRI, depending on their activation stage and phenotype [13].

Asiaticoside, a traditional Chinese medicine, is one of the primary active compounds derived from Centella asiatica (L.) Urban [14]. Previous studies have revealed that asiaticoside has numerous beneficial effect, including anti-inflammatory and anti-allergic, osteoclastogenesis, and immunoregulation effects [15–17]. Moreover, asiaticoside plays a critical role in chronic renal fibrosis, podocyte damage and diabetic nephropathy by regulating the expression of cytokines and the renal immune response [18–20].

However, few studies have evaluated the effects of asiaticoside on acute kidney injury.

This study aims to investigate the protective role of asiaticoside on renal ischemia-reperfusion injury in IRI model mice and its regulation of macrophage polarization in LPS-treated mouse macrophage cell line. In the present study, we hypothesize that asiaticoside protected kidney from IRI, which may be because of the inhibition of inflammatory mediators and promoting transformation of macrophages from M1 type to M2 type.

## 2. Materials and methods

### 2.1 Reagents

Asiaticoside (purity, ≥ 98%)was purchased from Nakeli biotech, Chengdu, China, whereas LPS was obtained from Sigma Aldrich, USA. ELISA kits for IL-1β (cat.no. PI301), IL-6 (cat.no. PI326), Interleukin-10 (IL-10) (cat.no. PI523), and Tumor Necrosis Factor-α (TNF-α) (cat.no. PT512) were purchased from Beyotime Biotechnology, Shanghai, China. Anti- iNOS (ab178945) and GAPDH (ab9485) were all obtained from Abcam, USA. Anti- Arginase1 (DF6657) and Goat Anti-Rabbit IgG (H + L) HRP (S0001) were procured from Affinity-Antibody, USA. F4/80 (71,299) and Anti-rat IgG (H + L), (Alexa Fluor® 488 Conjugate, 4416) were got from Cell Signaling Technology (CST), USA.

### 2.2 Cell culture

RAW264.7, a mouse macrophage cell line (Procell Life Science&Technology Co., Ltd, Wuhan, China), was cultured in DMEM high-glucose medium (HyClone, Logan, UT, USA) supplemented with 10% fetal bovine serum, 100 U/mL penicillin, and 100 μg/mL streptomycin at 37°C and 5% CO_2_.

### 2.3 Animal studies

6-week-old male C57BL/6 mice were purchased from Beijing Vital River Laboratory Animal Technology Co., Ltd. The mice were placed at 24 ± 0.5°C, 12/12 h light/dark cycle. All animal texting procedures were approved by Zhejiang University Animal Care and Use Committee.

Acute ischemic-reperfusion kidney injury (IRI) was induced in both kidneys as previously reported [4]. Briefly, 8-week-old male C57BL/6 mice were anesthetized by intraperitoneal injection of sodium pentobarbital (30 mg/kg body weight), followed by an incision, 1–5 cm long, at the back of the mouse. Next, both renal pedicles were clamped for 25 min. Notably, mice in the Sham groups underwent the same surgical procedure but without clamping of kidney pedicles. IRI mice were randomly divided into a low dose group (20 mg/kg), a medium dose group (40 mg/kg) and a high dose group (60 mg/kg) [21]. Saline or asiaticoside were administered via intraperitoneal injection after reperfusion for 6 h. Next, the above drugs were given once a day until the detection time (0, 1d, 3d and 7d). Blood samples were obtained after anesthesia, allowed to stand at room temperature for 30 min, and centrifuged at 3000 rpm for 10 min at 4°C to obtain serum. The kidney tissue was collected after anesthesia, followed by storing at −80°C. These samples were collected at the detection time for ELISA and biochemical analyses.

### 2.4 Serum biochemical measurements

Blood was centrifuged to obtain serum, followed by storing at −80°C until further analysis. The levels of BUN and Scr were determined using a biochemistry automatically analyzer (Hitachi 7020, Japan).

### 2.5 Western blot analysis

RAW264.7 cells were treated with LPS (100 μg/ml) or LPS (100 μg/ml) + asiaticoside (100 μM) for 48 h. Next, cells were lysed with RIPA lysis buffer (Beyotime, Shanghai, China) for 20 min at 4°C to extract total protein. Equal amounts (40 µg) of protein lysates were resolved by SDS-PAGE on 10% gels, and then transferred to a PVDF membrane. After blocking with 5% nonfat dry milk in PBS solution containing 0.02% (v/v) Tween-20, membranes were incubated with the primary antibodies (all 1:1000) at 4°C overnight. On the next day, membranes were washed and incubated with a peroxidase-labeled secondary antibody (1:5000) at room temperature for 1 hour. After washing, the protein bands were visualized by electrochemiluminescence (FD8030, FDBio Science, China).

### 2.6 Enzyme-linked immunosorbent assay (ELISA)

RAW264.7 cells were treated with LPS (100 μg/ml) or LPS (100 μg/ml) + asiaticoside (10, 25, 50, 100 μM) for 48 h [22], followed by collection of cell supernatants. Levels of IL-6, IL-1β, IL-10, and TNF-α were then determined using ELISA kits (Beyotime, Shanghai, China).

### 2.7 Immunofluorescence staining

For immunofluorescence, formalin-fixed slides were deparaffinized and antigen retrieval was performed by microwave oven heating. Sections were blocked with goat serum for 30 min in room temperature. Slides were incubated with primary antibodies (F4/80) overnight at 4  C. Secondary antibody (Anti-rat IgG (H + L)) was applied for 1 h at 37°C, following by DAPI for 5 min. Immunofluorescence was assessed using an Olympus BX53/DP80 microscope (Olympus Corporation, Shinjuku, Japan).

### 2.8 Statistical analysis

All statistical analyses were performed using SPSS version 22.0 software. All experiments were performed in triplicate and data were presented as means ± standard deviations. One-way analysis of variance (ANOVA) was used to compare multiple groups, whereas Student’s t test was used to compare two groups. P values <0.05 were considered statistically significant.

## 3. Results

Our study explored the effects of asiaticoside on renal IRI and its underlying mechanism, and found that asiaticoside protected the kidney injury mediated by IRI. The protective effect was related to the inhibition of inflammatory mediators and transformed M1 and M2 marker expressions of Raw264.7 cells.

### 3.1 Asiaticoside alleviated renal function in renal IRI mice

**Figure 1.**
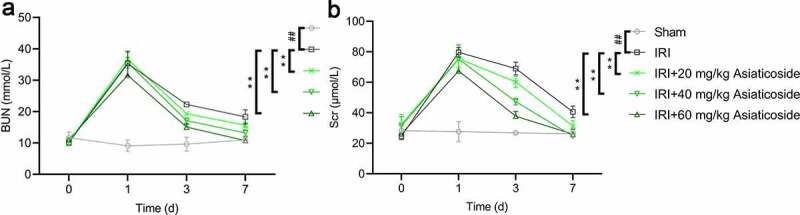
Asiaticoside alleviated renal function in IRI mice. BUN (a) and Scr (b) in renal ischemia-reperfusion injury mice, followed by saline or asiaticoside injection at 0, day1, day3, or day7. **p < 0.01 vs IRI groups, ##p < 0.01 vs Sham group. (n = 5 in each group).

An ischemic renal injury mouse model was established by clamping the bilateral renal pedicle for 25 min. Results showed that BUN and Scr levels were significantly increased in IRI groups at day 1 compared to the Sham group (Figure 1(a, b)), indicating that the IRI model mice were successfully established. Next, IRI mice were administered with saline or different doses of asiaticoside to explore the effects of asiaticoside. We found that the levels of BUN and Scr were almost recovered within 48 h in the asiaticoside-treated groups in a dose-dependent manner (Figure 1(a, b)). Collectively, these findings indicated that asiaticoside can promote renal function in IRI mice.

### 3.2 Asiaticoside inhibited the inflammation in renal ischemia-reperfusion injury mice

**Figure 2.**
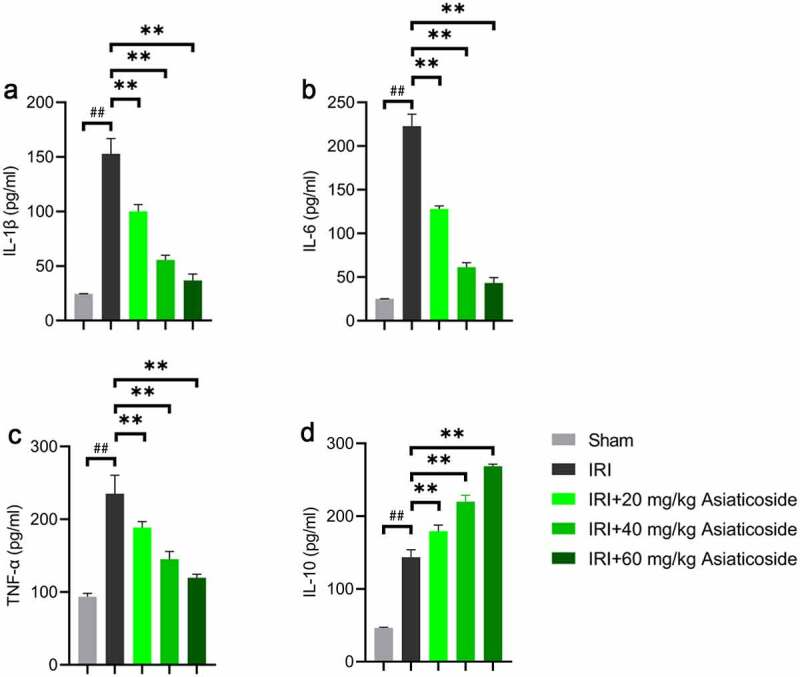
Asiaticoside inhibited the inflammation in renal ischemia-reperfusion injury mice. The levels of IL-1β (a), IL-6 (b), TNF-α (c), and IL-10 (d) in Sham group, IRI group, IRI with a low dose group (20 mg/kg), a medium dose group (40 mg/kg) and a high dose group (60 mg/kg) groups. **p < 0.01 vs IRI groups, ##p < 0.01 vs Sham group. (n = 5 in each group).

To investigate the functions of asiaticoside on the inflammatory response in IRI mice, we examined the inflammatory cytokine expressions using ELISA. Results indicated that renal ischemia-reperfusion injury significantly elevated the levels of IL-1β (Figure 2(a)), IL-6 (Figure 2(b)), TNF-α (Figure 2(c)), and IL-10 (Figure 2(d)) in the kidney compared to Sham groups. After asiaticoside intervention, the levels of IL-1β (Figure 2(a)), IL-6 (Figure 2(b)), and TNF-α (Figure 2(c)) were obviously alleviated. However, asiaticoside treatment further enhanced the expression of IL-10 (Figure 2(d)) induced by ischemia-reperfusion injury.

### 3.3 Asiaticoside reduced the infiltration of macrophages in renal IRI mice

**Figure 3.**
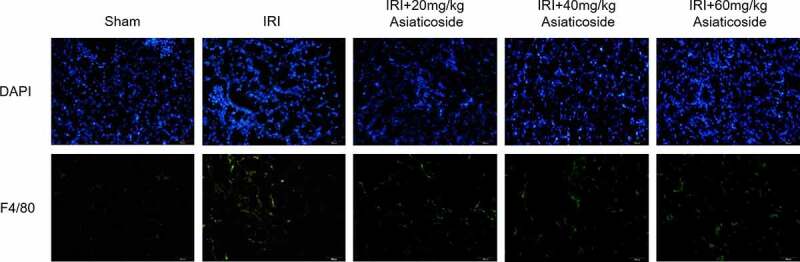
Asiaticoside reduced the infiltration of macrophages in renal IRI mice. The fluorescence intensity of F4/80 in Sham group, IRI group, IRI with a low dose group (20 mg/kg), a medium dose group (40 mg/kg) and a high dose group (60 mg/kg) groups.

The effect of asiaticoside on the infiltration of macrophages in kidney tissue of IRI model mice was detected by immunofluorescence staining (Figure 3). The fluorescence distribution of F4/80 in the kidney tissue of IRI model mice was significantly enhanced compared to Sham groups. The results showed that macrophage infiltration was increased in the kidney tissue of IRI model mice. Surprisingly, asiaticoside treatment could reduce the fluorescence intensity of F4/80 in kidney tissue. It indicated that asiaticoside inhibited the infiltration of macrophages in the kidney tissue of IRI model mice.

### 3.4 Asiaticoside attenuates LPS-induced inflammation in Raw264.7 cells

**Figure 4.**
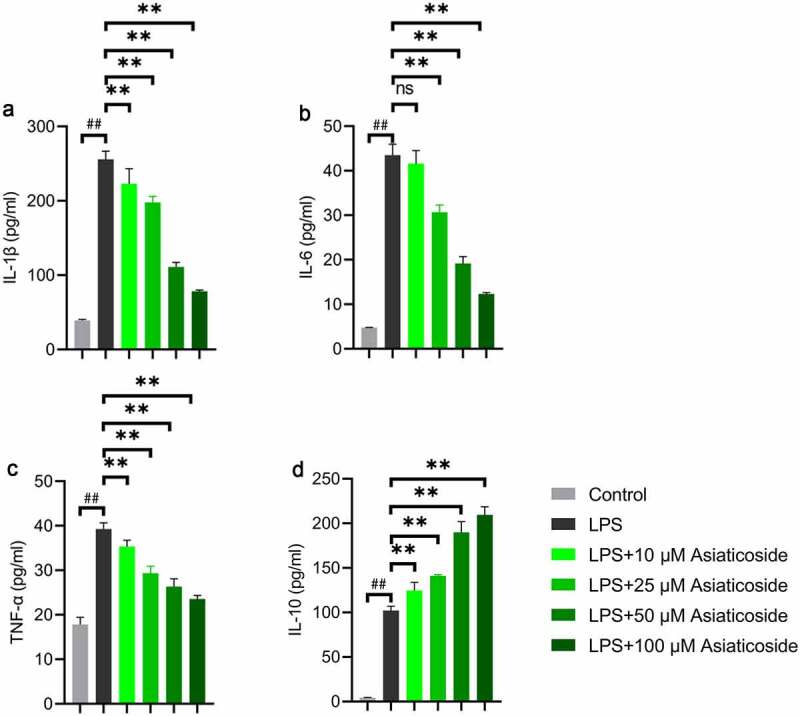
Asiaticoside attenuates LPS-induced inflammation in Raw264.7 cells. The content of IL-1β (a), IL-6 (b), TNF-α (c), and IL-10 (d) in the culture medium of Raw264.7 cells of different groups (control group, LPS group and LPS with different doses of asiaticoside). **p < 0.01 vs LPS groups, ##p < 0.01 vs control group.

The levels of IL-1β (Figure 4(a)), IL-6 (Figure 4(b)), TNF-α (Figure 4(c)), and IL-10 (Figure 4(d)) inflammatory factors were higher in the LPS group compared to the control group. However, the levels were reduced by asiaticoside. In contrast, the level of IL-10 (Figure 4(d)) was higher in the asiaticoside treatment groups than in the LPS group. These findings suggest that asiaticoside inhibited the inflammation induced by LPS in Raw264.7 cells.

### 3.5 Asiaticoside improved LPS-induced inflammation and transformed M1 and M2 marker expressions in Raw264.7 cells

**Figure 5.**
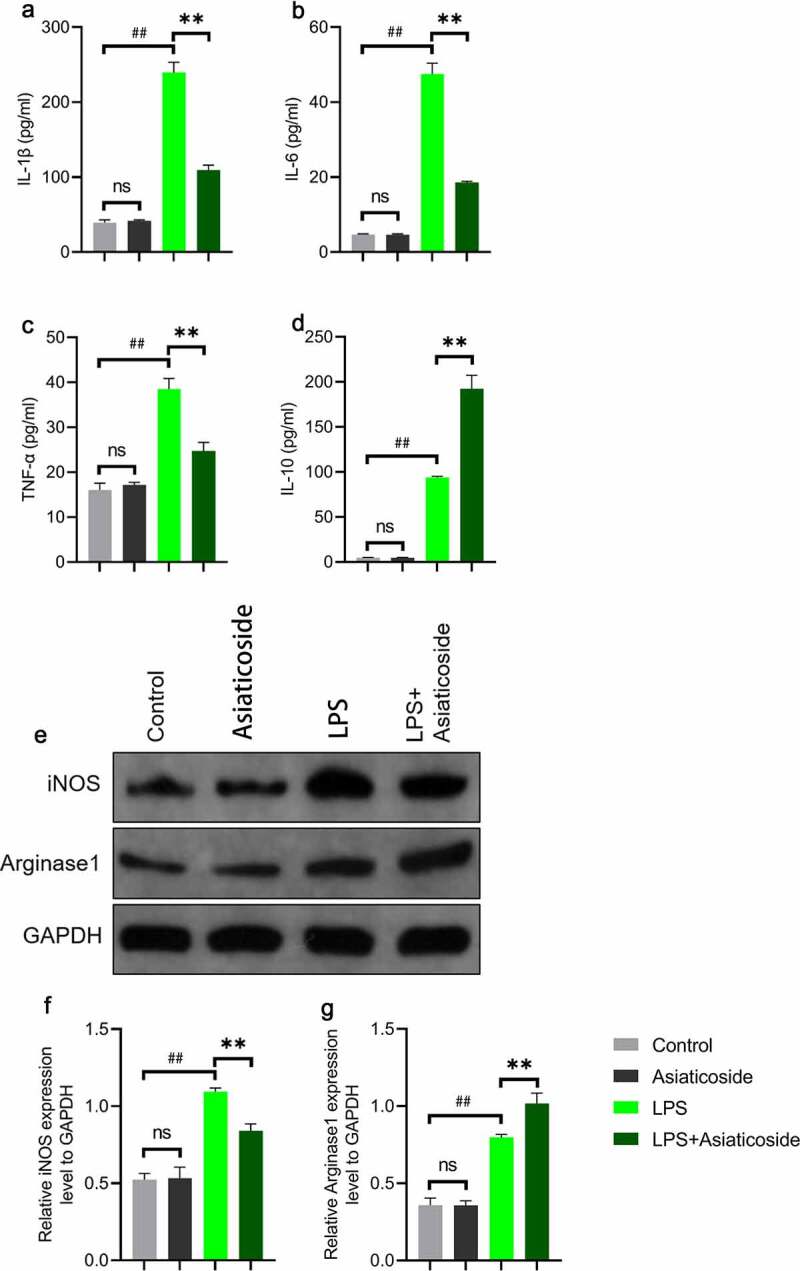
Asiaticoside improved LPS-induced inflammation and transformed M1 and M2 marker expressions in Raw264.7 cells. The levels of IL-1β (a), IL-6 (b), TNF-α (c), and IL-10 (d) in the culture medium of Raw264.7 cells of different groups (control group, asiaticoside (100 μM) group, LPS group and LPS+ asiaticoside (100 μM) group). (e)After immunoblotting, the levels of iNOS and Arginase1 were identified using their specific antibodies. The expression s of iNOS (f) and Arginase1 (g) are demonstrated. **p < 0.01 vs LPS group, ##p < 0.01 vs control group, nsp > 0.05 vs control group.

There was no change in the secretion of inflammatory factors Raw264.7 cells under asiaticoside treatment alone compared to the control group (Figure 5). Treatment with 100 μM asiaticoside attenuated the activation of IL-1β (Figure 5(a)), IL-6 (Figure 5(b)) and TNF-α (Figure 5(c)), but LPS-treated cells increased IL-10 from the control and asiaticoside augmented this increase.

To further clarify the mechanism underlying the effects of asiaticoside on LPS-induced Raw264.7 cells, western blot analysis was performed to elucidate the potential pathways. Results showed that LPS increased the expression of M1 marker (inducible NO synthase-iNOS) and M2 marker (Arginase1) in Raw264.7 cells (Figure 5(e)). After asiaticoside treatment, the expression of iNOS (figure 5(f)) was suppressed, but the expression of Arginase1 (Figure 5(g)) was further improved. This suggested that asiaticoside ameliorated the inflammatory response induced by LPS through M2 polarization.

## 4. Discussion

This study found that asiaticoside ameliorated the increased levels of serum BUN and Scr, as well as inflammatory factors, induced by renal ischemia-reperfusion injury. Moreover, Raw264.7 cells with LPS-induced inflammation, exhibited enhanced inflammatory mediators, including IL-1β, IL-6, TNF-α, and IL-10, but asiaticoside did not attenuate the increase in IL-10. In addition, asiaticoside treatment induced Raw264.7 cells toward M2 polarization by regulating M1 and M2 markers.

It is well known that IRI is a systemic inflammatory disease. A previous study reported that the repair of renal IRI is associated with the production and release of pro-inflammatory and anti-inflammatory factors [23]. Cellular mediators of immunity, such as macrophages, neutrophils, natural killer T, T and B cells, contributes to the pathogenesis of renal injury and repair after IRI [24]. For example, IL-10 is a pleiotropic cytokine, secreted by a variety of immune cells including regulatory T cells, renal tubular epithelial cells, and helper T cells [25]. IL-10 modulates the innate and adaptive immune responses, through exerting immunosuppressive functions to reduce tissue damage [26]. Meanwhile, the regulatory immune cell network is activated by the mutual regulation of TNF-α and IL-10 to maintain immune homeostasis after IRI [27]. TNF-α can induce a variety of inflammatory gene expression, thereby increasing inflammation [28]. These studies confirm our findings, and indicate that the secretion of IL-10 and TNF-α was stimulated by LPS in macrophages. However, asiaticoside treatment attenuated IRI-induced inflammation by adjusting the expression of inflammatory mediators.

Macrophages are involved in both inducing renal damage and promoting renal repair after renal IRI [27]. On one hand, M1 macrophages express high levels of inducible nitric oxide (NO) synthase and proinflammatory mediators such as IL-6, TNF-α and IL-12 [29]. On the other hand, M2 macrophages tend to promote wound healing and inflammation resolution by inducing IL-4 and IL-13, which are anti-inflammatory factors [30].

NO synthases (NOS) isoforms synthesize NO, a small signaling molecule. Among them, the third NOS isoform (iNOS) is negligible in resting cells and stimulated by cytokines and bacterial LPS [31]. Arginase1 is one of members of the arginase family [32]. Differential metabolism of L-arginine is characteristic of M1 and M2 macrophages by iNOS and Arginase1 [33]. The M1 and M2 macrophage activation stems from exactly these alternative metabolic states reflected by iNOS/Arginase1 balance. Recent evidences suggest that Arginase1 plays a fundamental role for M2 macrophages to acquire the phenotype [34]. In the future study, we will use the blockers and interference to investigate the exact molecular mechanism by which asiaticoside promotes the transformation of macrophages.

Surprisingly, the expression of iNOS-M1 marker was suppressed after asiaticoside treatment, but the expression of Arginase1-M2 marker was further improved. Collectively, these findings suggest that asiaticoside played a role on the polarization state of macrophages to some extent. However, further studies should be conducted to discover the molecular mechanism of asiaticoside on macrophage polarization regulation, and these results should also be verified in human disease.

## 5. Conclusion

In conclusion, the findings of this study provide evidence for the protective effects of asiaticoside on IRI mice and LPS-stimulated inflammatory responses in Raw264.7 cells. Furthermore, the results demonstrated the therapeutic potential of asiaticoside in renal ischemia-reperfusion injury.
